# The working angle in low-abrasive air polishing has an influence on gingival damage—an ex vivo porcine model

**DOI:** 10.1007/s00784-023-05236-3

**Published:** 2023-08-29

**Authors:** Jens Weusmann, James Deschner, Christopher Keppler, Jean-Claude Imber, Pablo Cores Ziskoven, Sven Schumann

**Affiliations:** 1 Department of Periodontology and Operative Dentistry, University Medical Center of the Johannes Gutenberg-University Mainz, Augustusplatz 2, 55131 Mainz, Germany; 2 Department of Periodontology, University of Bern, Freiburgstrasse 7, 3010 Bern, Switzerland; 3 Institute of Anatomy, University Medical Center of the Johannes Gutenberg-University Mainz, Langenbeckstraße 1, 55131 Mainz, Germany

**Keywords:** Low abrasive air polishing, Porcine ex vivo model, Tagatose, Glycine, Working angle, Gingiva

## Abstract

**Objectives:**

To investigate the influence of instrumentation angle during low-abrasive air polishing (LAA) on the oral gingiva using an ex vivo porcine model.

**Material and methods:**

Six tissue samples from each of 14 porcine mandibles were randomly selected and instrumented. Two different LAA powders (glycine 25 μm, tagatose 15 μm) were investigated. An application angle of either 30–60° or 90° was selected. Gingival specimens from different mandibles served as untreated references. Gingival biopsies were examined by scanning electron microscopy and paraffin histology for tissue destruction using a five-level scale.

**Results:**

LAA caused significantly less tissue damage at a 90° angle than at a 30–60° angle. This effect was seen in both the glycine-based powder arms (*p* = 0.002, *p* = 0.046) and the tagatose-based powder arms (*p* = 0.003, *p* = 0.011). However, at identical working angles, the two powders did not show significant differences in terms of gingival erosion (*p* = 0.79 and *p* = 0.57; *p* = 0.91 and *p* = 0.78, respectively).

**Conclusions:**

LAA may cause less tissue damage at an application angle of 90°. Consequently, it seems advisable to air-polish the soft tissue as perpendicularly as possible. Additionally, glycine and tagatose LAA powders do not seem to differ in concern of soft tissue damage.

**Clinical relevance:**

Within the limitations of this ex vivo animal model, this study argues for an application that is as close as possible to the 90° angle intending to minimize soft tissue damage. Manufacturer specifications, however, mainly request applications deviating from the right angle. In order to work in interdental areas using LAA safely, the use of subgingival nozzles might be considered.

## Introduction

Periodontitis and gingivitis are caused by the presence of a pathogenic oral biofilm [1–5]. In reversible plaque-induced gingivitis, removal of dental plaque is the treatment of choice. Periodontitis, which can be considered associated with oral biofilm dysbiosis, is classified into different stages and grades [6, 7]. Independently of the disease severity, disintegration and, if possible, elimination of the dental biofilm play an important role in all four therapy steps of periodontitis therapy in the context of supragingival and subgingival debridement [8, 9]. The first step of the periodontal therapy includes the supragingival plaque removal, the second step includes supra‐ and subgingival instrumentation, the third step comprises surgical interventions, and the fourth step relates to supportive periodontal care (SPC) [8, 10].

In SPC, periodontal pockets and supragingival plaque are often continuously cleaned. In this context, both periodontal soft tissues and dental hard tissues showed significant substance damage by ultrasonic scalers and hand instruments after repeated use [11–14].

As an alternative, low-abrasive air polishing (LAA) was developed to facilitate biofilm removal while allowing tissue-sparing debridement. It has been shown that up to a pocket depth of 5 mm, this procedure is more effective than hand instruments in removing subgingival plaque [15–17]. Periodontal pockets 5 mm to 9 mm can be cleaned by glycine LAA using a subgingival nozzle more effectively than by manual or ultrasonic instruments [18]. After the introduction of LAA with glycine, several other powders based on non-cariogenic sugars were established.

Potential adverse effects of air polishing on oral tissues had been addressed previously [19–22]. In all mentioned studies, highly abrasive powders were used. It has been revealed that glycine powder air polishing is less harmful to oral soft and hard tissues [22, 23]. However, the effect of LAA powders in direct comparison and the effect of differences in the working angle have been scarcely studied.

Different manufacturer specifications exist regarding the recommended angle of low-abrasive powder jet application. The following study examines differences between the 30–60° angle recommended in instructions for use and the only partially recommended 90° angle using a long-established glycine-based powder and a novel tagatose powder.

The aim of this study was to evaluate the influence of different application angles on keratinized gingiva in a porcine ex vivo model. It was hypothesized that the LAA working angle has no influence on gingival damage.

## Material and methods

### Instrumentation and biopsy

Porcine mandibles were obtained from a nearby abattoir and stored for no longer than 6 h post-mortem and at a constant temperature of 7 °C. The mandibles were fixed in kidney dishes, which allowed controlled instrumentation.

A total number of 84 samples were collected from 14 mandibles. Four variants of LAA and controls were investigated.

The glycine powder used was EMS Perio Powder (EMS, Nyon, Switzerland) with a mean particle size of 25 μm.

The tagatose powder used was smartPearls plus Powder (smartdent, Rodgau Nieder-Roden, Germany) with a mean particle size of 15 μm.

**Fig. 1:**
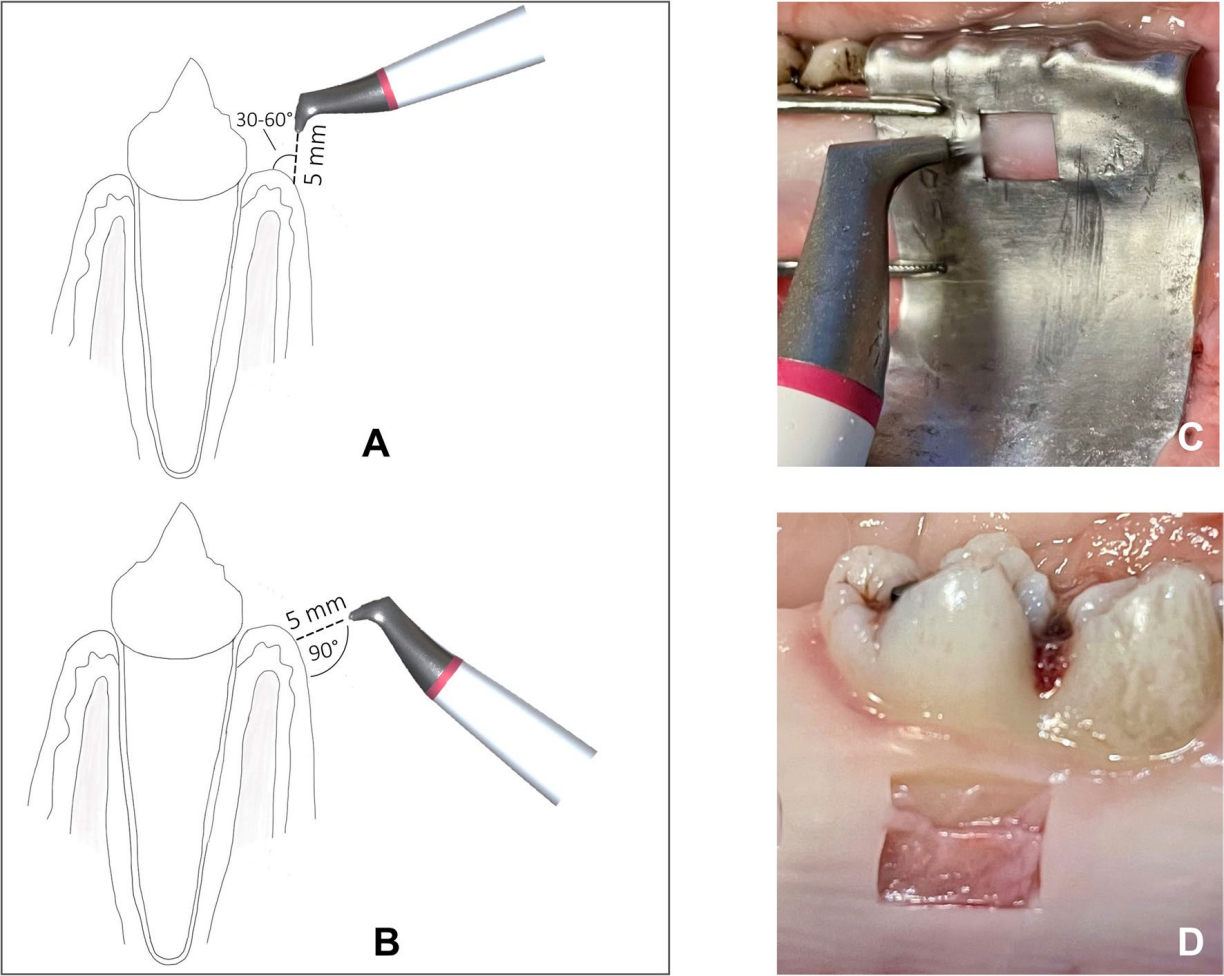
Experimental procedure. Schematic representation of the LAA with an angle of 30–60° (**A**) or 90° (**B**). Surrounding tissue was covered with a template made of tin foil (**C**). After LAA, the treated area was removed for morphological analysis (**D**)

An “EMS Air Flow Master” air polishing device (EMS) with a standard handpiece for supragingival LAA was used in all groups. Instrumentation was performed using an adaptable template made of tin foil with a window to avoid damaging the surrounding tissue and to obtain accurate, comparable specimens (Fig. 1A). The handpiece nozzle was kept at a distance of 5 mm from the gingival tissue at all times, and the powder beam was directed at the gingiva for 5 s with slow, rotating movements intending to instrument the entirety of the specimen. Maximum water and powder flow were always used for the examinations. Either a 30–60° angle (Fig. 1A) or a 90° angle (Fig. 1B) to the surface of the gingiva was used.

After LAA application, the treated gingiva was removed using a type 15 blade, always separated deeply in the connective tissue (Fig. 1D). Six gingival samples were collected from each porcine jaw.

Twenty specimens per study arm and four untreated control specimens were fixed in buffered formaldehyde solution (4%). For scanning electron microscopy (SEM), tissue samples (eight per study arm and two controls), as well as samples of the used powders, were dehydrated in ethanol, freeze-dried, sputtered with gold in an argon atmosphere, and visualized with a scanning electron microscope (ESEM XL-30, Philips, Eindhoven, The Netherlands). For histological evaluation, hematoxylin and eosin (H&E) staining was performed after paraffin embedding of the samples (twelve per study arm and two controls). Seven-micrometer sections were deparaffinized in xylene and rehydrated in ethanol with decreasing concentration. H&E staining was performed with hematoxylin (Sigma-Aldrich, St. Louis, Missouri, USA) and eosin (Sigma-Aldrich) after rinsing with distilled water. Staining was followed by dehydration with ethanol at increasing concentrations and treatment in xylene. Histological sections were photographed using a Leica MS 5 tripod (Leica Microsystems, Germany) and a JVC KY-F75U C-mount digital camera (JVC, Yokohama, Japan). A five-level scoring system was used for both histology and SEM (Table 1).

**Table 1:** Five-level scoring system for the assessment of soft tissue damage

Tissue damage	Score
No damage	0
Superficial epithelial damage	1
Deeper epithelial damage	2
Epithelial detachment	3
Connective tissue damage	4

### Statistical analysis

Statistical analysis was performed using the chi-square test (MS Excel, Microsoft, Redmond, USA, GraphPad Prism 8.0, San Diego, USA). *P*-values < 0.05 were defined as statistically significant.

## Results

### SEM

**Fig. 2:**
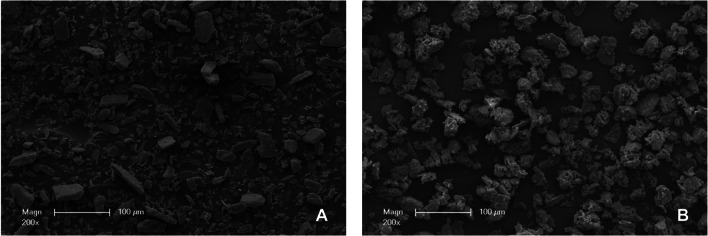
Representative scanning electron microscopy images of the used powders (EMS Perio powder (**A**), smartPearls plus powder (**B**))

**Fig. 3:**
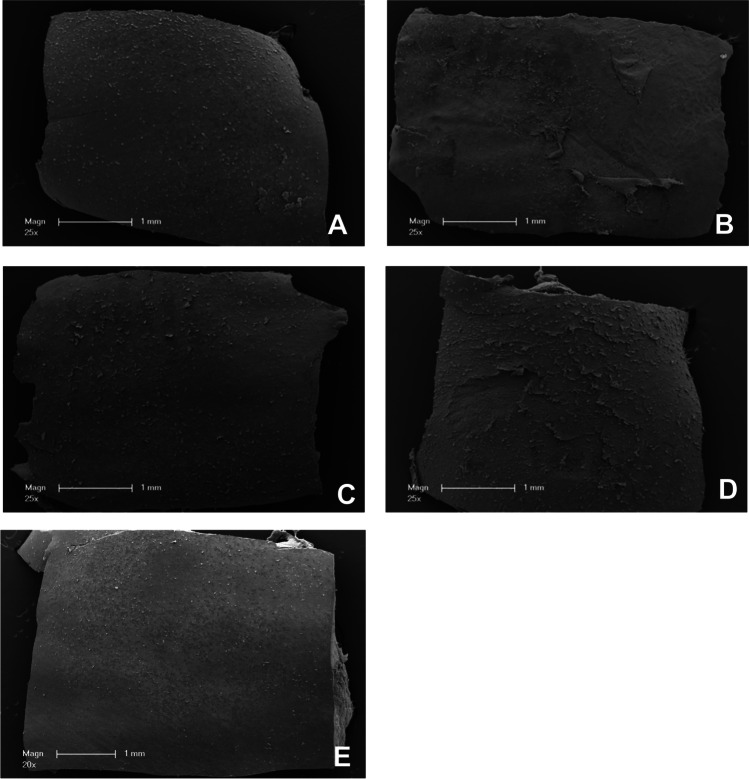
Representative scanning electron microscopy images of gingiva samples after treatment. **A** Treatment with EMS Perio at an angle of 90°. **B** Treatment with EMS Perio at an angle of 30–60°. **C** Treatment with smartPearls plus at an angle of 90°. **D** Treatment with smartPearls plus at an angle of 30–60°. **E** Control without treatment

**Fig. 4:**
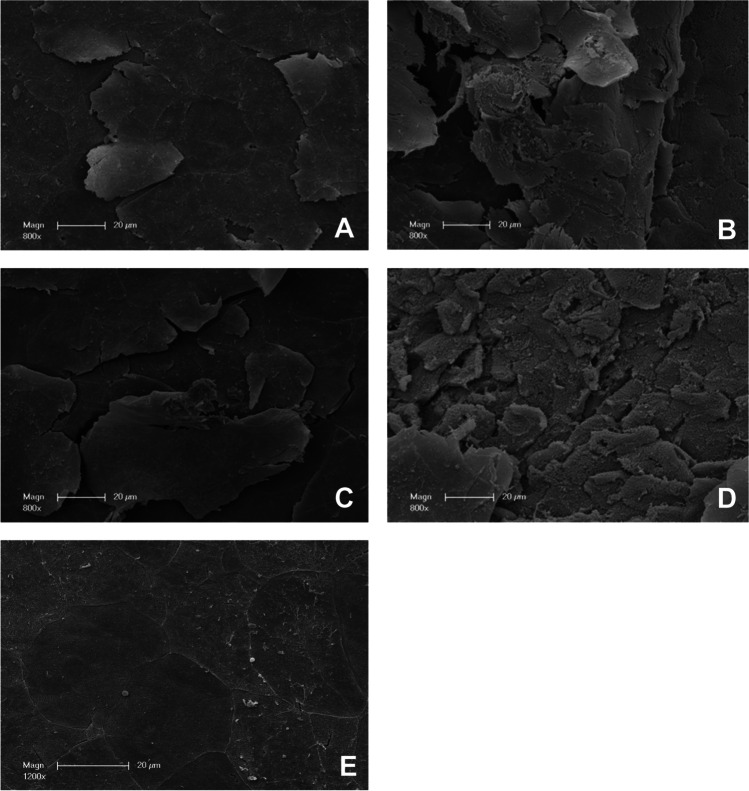
Representative scanning electron microscopy images of gingiva samples after treatment. **A** Treatment with EMS Perio at an angle of 90°. **B** Treatment with EMS Perio at an angle of 30–60°. **C** Treatment with smartPearls plus at an angle of 90°. **D** Treatment with smartPearls plus at an angle of 30–60°. **E** Control without treatment

Examination of the SEM images of the two powders used revealed greater heterogeneity of visible grain sizes for glycine powder (Fig. 2A) than for tagatose powder (Fig. 2B). In contrast, the surface of tagatose powder appeared rougher and more furrowed than that of glycine powder. Untreated gingiva showed an intact keratinized surface (Figs. 3E and 4E).

In the glycine powder, 90° group (Gly 90), an intact epithelium was visible in all cases (Figs. 3A and 4A). In several cases, the margins of the horn scales of whole scales seem to be detached. In the glycine powder, 30–60° group (Gly 30–60), epithelial defects down to the underlying connective tissue could be seen (Figs. 3B and 4B). In the tagatose powder, 90° group (Tag 90), an intact epithelium with detached horn scales, was visible (Figs. 3C and 4C), while in the tagatose powder, 30–60° group (Tag 30–60) the submucous layer was partially exposed (Figs. 3D and 4D).

**Fig. 5:**
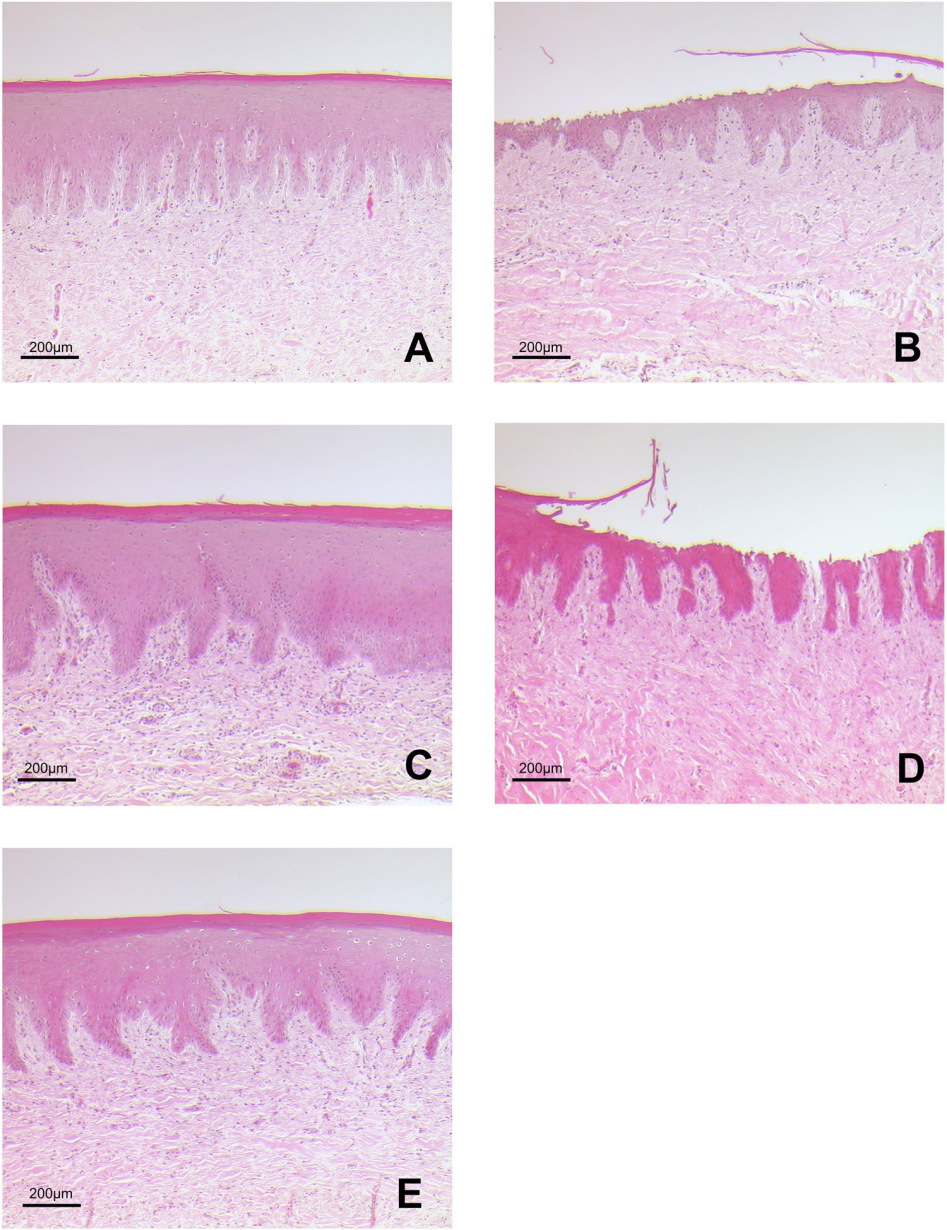
Representative scanning electron microscopy images of gingiva samples after treatment. **A** Treatment with EMS Perio at an angle of 90°. **B** Treatment with EMS Perio at an angle of 30–60°. **C** Treatment with smartPearls plus at an angle of 90°. **D** Treatment with smartPearls plus at an angle of 30–60°. **E** Control without treatment

**Fig. 6:**
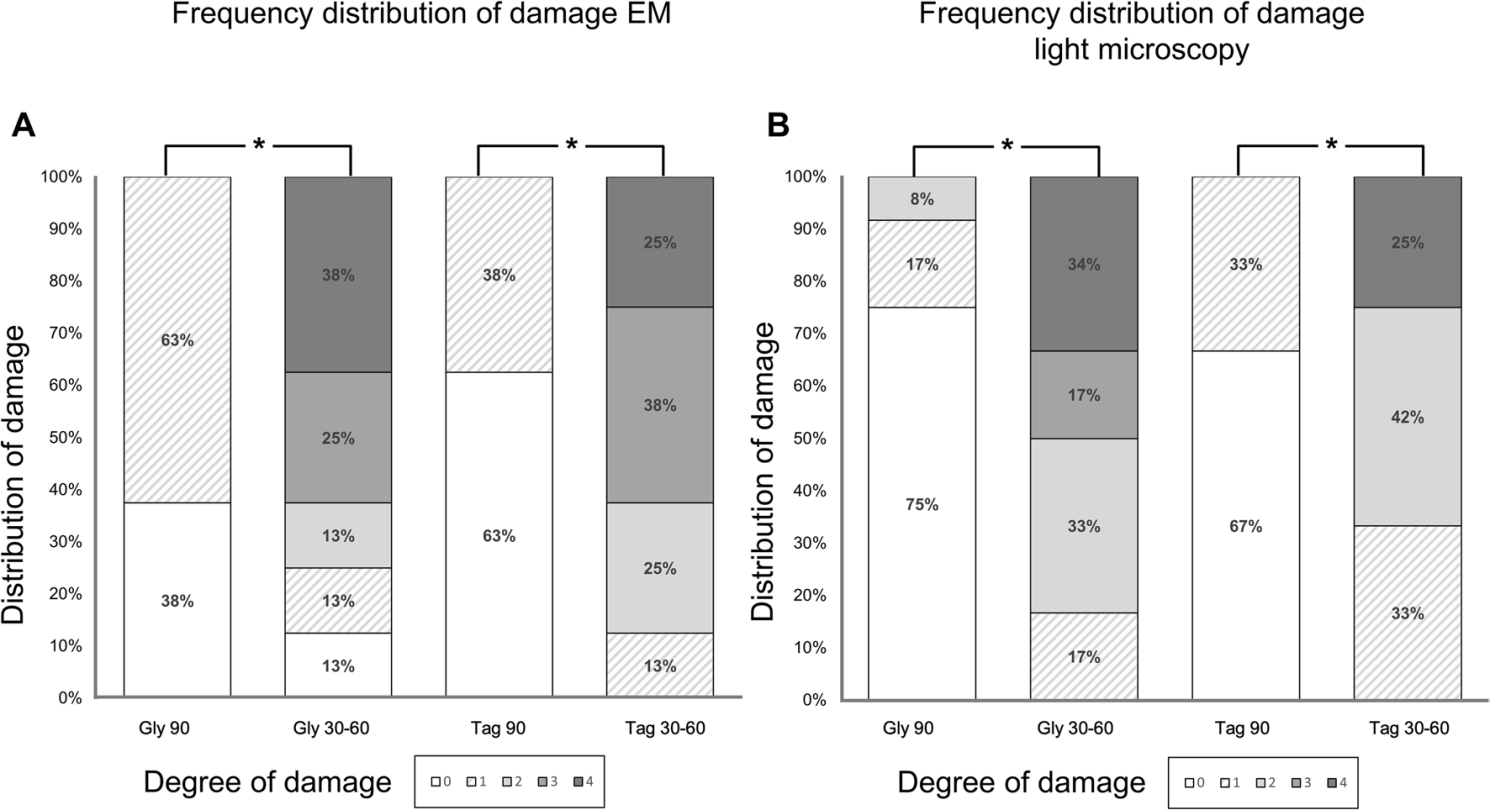
Stacked bar diagram showing the distribution of tissue damage scores within the groups. For SEM evaluation, eight specimens per group were evaluated. Twelve specimens were analyzed for light microscopy. Statistics performed by chi-square test, *p < 0.05

Regarding the frequency of samples with a score of 2 to 4, treatment at an angle of 30–60° resulted in more pronounced tissue damage (Gly 30–60: 75%; Tag 30–60: 88%) independently of the powder. No sample in Gly 90 and Tag 90 was classified as 2, 3, or 4. In total, 38% of the samples in Gly 90 were classified as undamaged, while 63% received a score of 1. In Gly 30–60, there was only one sample with no damage, 13% of the samples had a score of 1 or 2 with a score of 3, 25% of the samples were classified, and the other 38% received a score of 4. Overall, 63% of the samples at Tag 90 did not show any damage, and the remaining 38% had a damage score of 1. In Tag 30–60, 13% of the samples were assessed a score of 1, 25% of the samples received a score of 2, 38% were classified with a score of 3, and the remaining 25% of the samples received a score of 4 (Figs. 5 and  6A). The injurious effect was significantly less pronounced in Gly 90 than in Gly 30–60 (*p* = 0.046). The situation was analogous for Tag 90 compared to Tag 30–60 (*p* = 0.011).

### Histology

Untreated gingiva showed a normal histological structure with a well-developed corneal layer on the surface. In Gly 90, an intact gingival epithelial surface with a minor detachment of horn scales and small clefts within the corneal layer were visible in most cases (Fig. 5A). Samples of Gly 30–60 showed deep lesions which could reach the connective tissue (Fig. 5B). In Tag 90, an almost undamaged gingiva is presented showing slight exfoliation of the keratinized scales (Fig. 5C). In contrast, deep epithelial lesions which dominate in Tag 30–60 (Fig. 5D).

Soft tissue damage was significantly less pronounced in Gly 90 than in experimental Gly 30–60 (*p* = 0.002). A similar observation was seen in Tag 90 compared to Tag 30–60 (*p* = 0.003). Concerning experimental Gly 90 and Tag 90 as well as Gly 30–60 and Tag 30–60, there was no significant difference (*p* = 0.79 and *p* = 0.57, respectively) (Fig. 6B).

As shown by the frequency of samples with a score of 2 to 4, treatment with an angle of 30–60° caused significant tissue damage largely independent of the powder (Gly 30–60: 83% and Tag 30–60: 67%). None of the samples in Gly 90 and Tag 90 were given a damage score of 3 or 4, nor was only one sample given a damage score of 2. Overall, 75% of the samples in Gly 90 were rated as undamaged while 17% received a damage score of 1, with only one sample (8%) receiving a damage score of 2. In Gly 30–60, there was no sample without damage, 17% of the samples had a damage score of 1, and the other 67% were equivalently distributed between damage scores 2 and 4. Altogether, 67% of the samples in Tag 90 exhibited no damage, and the other 33% exhibited damage score 1. In Tag 30–60, 33% of the samples were given a damage score of 1; 4% of the samples were given a damage score of 2, and the remaining 25% of the samples were given a damage score of 4 (Fig. 6B).

## Discussion

In this ex vivo study, two different LAA powders and two different working angles were investigated. It has been found that different working angles produced different degrees of soft tissue damage; thus, the null hypothesis could be rejected. In that concern, the damage was significantly greater when a working angle of 30–60° was used compared to the use of a 90° working angle. Consequently, the central powder beam should always be applied to the soft tissue as close as possible to the 90° angle. It should be mentioned, however, that the underlying experiment is an ex vivo model, which is why the question of clinical applicability remains unanswered.

Several studies had already been conducted investigating air polishing’s impact on oral soft tissue [19–23]. It had been found that air-polishing using powders based on sodium bicarbonate could cause severe damage to the gingiva. In comparison, LAA is far less harmful in this concern [23].

To our knowledge, the present study is the first to address the question of the least tissue damaging working angle on gingiva. The underlying ex vivo model proved to be suitable for comparing the impact of biofilm removal procedures and gingival tissues in previous work [24]. In the present study, attached gingiva was harvested slightly apical to the periodontal margin (Fig. 1). However, due to the localization of our sampling sites, a similar gingival condition could be expected as in the publication by Petersilka et al. Both sulcular epithelium and vestibular gingival epithelium are multilayered and similarly parakeratinized squamous epithelia. In our study, the areas to be instrumented by LAA were previously marked with a tin foil template and the tissue to be examined was cut out accordingly. Furthermore, our group decided to use a more diversified damage score, which classifies the observations into scores 0–4 (Table 1). Also in our study, the analysis of tissue damage was based on histological images (Fig. 5), but here, these were further supplemented by SEM images (Figs. 3 and 4). In the above study, additionally to two LAA powders, the effect of curettes and piezoelectric ultrasound scalers was also tested. Both procedures were found to be significantly more damaging to soft tissue than subgingival low-abrasive air polishing [24]. Similar to the study conducted by our research group, no significant differences were found between the LAA powders tested. Comparing the results of our ex vivo study to Petersilka et al., 2008, the damage pattern—at least when looking at the LAA at a 90° angle—is quite similar to the LAA powders used by Petersilka et al. [22]. However, a different picture emerges with the 30–60° LAA in our study: here, only a minority of the specimens showed undamaged, and more severe tissue damage occurred. In that study by Petersilka et al., 20% of the control group also showed mild damage. This could be explained by the slightly different sampling localization and the gingival margin spontaneously damaged by food intake. The glycine powder used in our study was identical to that used by Petersilka et al. 2018. In contrast, the other LAA powder used in our study was a comparatively novel tagatose-based powder. Tagatose LAA has been shown to have a good in vitro cleaning potential, as well as no negative impact on osteogenic differentiation on human dental pulp stem cells seeded on titanium disks [25]. We know from previous research that LAA powders can exert a direct effect on cell metabolism in human gingival cells in vitro [26–28]. The damage pattern similar to a glycine powder on porcine oral gingiva might be a first indication that tagatose could become a good alternative to other, currently better researched LAA powders.

A study that investigated different working angles on dental root tissue found no significant difference between 45° and 90° applications [13]. The negative effect of acute-angle machining that we discovered can be well explained by the comparatively lower resistance to tensile forces than to compressive forces in oral tissues. Since it is almost impossible to use LAA at a 90° angle at all times, especially in areas such as the interdental col, it would be advisable to use subgingival nozzles that divert the beam in this area. Although porcine gingiva closely resembles the human gingiva, there are differences. It would be desirable to be able to perform similar studies on human epithelium to confirm transferability. This would be possible on cadavers, but this approach has its limitations. It remains unclear to what extent other changes in parameters could be helpful. The present study used LAA parameters that had been used in the past. It would be questionable to what extent instrumentation time intervals and powder flow have a further influence. This study included only microscopic tissue examinations. It is known that LAA powders can influence viability, inflammation, and wound healing [26–28]. These were not investigated in the present study. A statement on the cleaning effectiveness, of course, cannot be derived from our study.

Within the limitations of an ex vivo porcine model study, our findings revealed an angle dependency concerning the soft tissue damage in LAA. One long-term established glycine powder showed similar results as a novel tagatose powder, despite distinct morphological differences. Apparently, the aim should be to instrument the oral gingiva at a 90° angle as close as possible when using LAA. It will be of interest to investigate to what extent the generally non-rectangular LAA instrumentation in interdental spaces plays a role. Further studies on human tissues would be of interest regarding transferability to humans.

## Data Availability

The data are available from the corresponding author upon request.
